# A Machine Learning-Derived Taurine Metabolism Signature Predicts Prognosis and Immune Landscape in Lung Adenocarcinoma via Integrative Single-Cell Analysis

**DOI:** 10.1155/mi/6610564

**Published:** 2025-11-25

**Authors:** Meng Wang, Qiuqiao Mu, Yuhang Jiang, Yuhao Jing, Yifan Zhao, Xingpeng Han

**Affiliations:** Tianjin Chest Hospital, Tianjin University, Tianjin, China

**Keywords:** immunotherapy, *KIF2C*, LUAD, machine learning, scRNA-seq

## Abstract

**Background:**

Lung adenocarcinoma (LUAD) represents a biologically diverse tumor type, often associated with unfavorable prognosis and unsatisfactory therapeutic outcomes. Over the past few years, increasing attention has been given to metabolic alterations as key contributors to cancer development. Nevertheless, the specific contribution of taurine-related metabolic pathways in LUAD remains unclear. By constructing a model from taurine metabolism-associated genes, we aimed to elucidate its mechanistic basis and evaluate its relevance to clinical outcomes.

**Methods:**

Transcriptomic profiles and clinical annotations from TCGA along with five LUAD datasets from the GEO repository were comprehensively integrated. A risk score indicative of taurine metabolism-associated signature (taurine-related signature [TRS]) was constructed by integrating LASSO regression, stepwise Cox modeling, and SuperPC algorithm. Its predictive capability was systematically evaluated using Kaplan–Meier survival analysis, ROC curves, and DCA. To further investigate the relationship between TRS and both cellular heterogeneity and tumor microenvironmental context, single-cell RNA-seq data were integrated into the analysis. Moreover, the tumorigenic role of the hub gene *KIF2C* was experimentally validated via in vitro functional assays.

**Results:**

The TRS signature was validated for its predictive relevance using six LUAD datasets from independent sources, showing its ability to categorize patients based on survival variations. Elevated TRS levels were strongly linked to increased tumor cell proliferation, immune evasion characteristics, and impaired response to immunotherapy. Findings from single-cell RNA sequencing indicated that epithelial subpopulations with higher TRS expression displayed intensified metabolic activity and reduced antigen presentation efficiency. In particular, *KIF2C*—a key component of the TRS gene set—was highly expressed in LUAD tissues and associated with less favorable prognostic profiles. Functional silencing of *KIF2C* led to decreased proliferation and invasive capacity of LUAD cells, supporting its potential role as a tumor-promoting factor.

**Conclusion:**

We established a taurine metabolism-related prognostic model (TRS) and investigated its function in LUAD by combining transcriptomic information from both bulk tissues and single-cell datasets. The TRS effectively categorizes patients according to their prognosis and reflects immune-related features. *KIF2C*, identified as a crucial gene within the TRS, could be explored as a potential therapeutic candidate, offering insights into LUAD metabolism and informing the development of metabolism-based therapeutic strategies.

## 1. Introduction

Among the various histological forms of non-small cell lung cancer (NSCLC), lung adenocarcinoma (LUAD) is the most prevalent and its incidence and associated disease burden continue to rise globally, especially in individuals with smoking history or environmental exposure [1, 2]. Although notable progress has been made in both diagnostic procedures and therapeutic interventions, the long-term survival rate for LUAD patients remains dismally low, typically under 20% [3, 4]. This malignancy exhibits pronounced heterogeneity, with wide-ranging differences across genomic alterations, immune landscape, and therapeutic sensitivity, complicating clinical decision-making [5]. These challenges underscore the importance of establishing reliable prognostic stratification systems to facilitate individualized treatment planning and improve overall clinical outcomes.

Conventional prognostic frameworks are frequently established by applying LASSO regression together with the Cox proportional hazards model [6]. Although these methods offer advantages in variable selection and survival prediction, their predictive stability and generalizability are often compromised in high-dimensional datasets due to issues such as overfitting and limited cross-cohort performance. Machine learning has recently gained prominence as an effective approach for uncovering intricate patterns embedded within high-dimensional datasets [7, 8]. Compared with conventional statistical models, machine learning approaches—such as random forest, support vector machines, SuperPC, and other ensemble-based algorithms—provide superior ability to capture complex variable interactions while improving predictive accuracy and model robustness [9–11]. These techniques have demonstrated promising results across a range of cancer types, providing new avenues for risk stratification and individualized treatment in LUAD.

The advent of scRNA-seq has remarkably advanced the exploration of cellular diversity underlying tumor heterogeneity [12, 13]. By capturing gene expression patterns at the individual-cell level, scRNA-seq allows in-depth characterization of subpopulations, intercellular communication, and immune ecosystem dynamics within the tumor microenvironment [14, 15]. The integration of scRNA-seq with computational modeling offers enhanced biological interpretability and predictive power, further advancing the development of clinically relevant prognostic signatures [16].

Taurine is a sulfur-based amino acid widely present across mammalian tissues and participates in key physiological processes, including bile salt formation, calcium balance, osmotic regulation, and cellular antioxidation [17, 18]. Recent studies indicate that taurine exhibits context-dependent functions in cancer, potentially exerting both pro- and antitumorigenic effects [19]. On the one hand, its antioxidant and antiapoptotic properties may support tumor cell survival [20, 21]; on the other hand, taurine appears to counteract tumor growth by interfering with mitochondrial metabolism and stimulating immune cell function [22, 23]. Nevertheless, the precise contribution of taurine metabolism–associated genes to LUAD has yet to be fully elucidated. Characterizing these metabolic processes could provide deeper understanding of tumor mechanisms and highlight candidate directions for therapeutic development.

Building on this rationale, this work conducted a comprehensive analysis of transcriptomic datasets from multiple LUAD cohorts, including TCGA and GEO, to evaluate the prognostic value of taurine metabolism–associated genes. Taurine-related signature (TRS) was constructed and its prognostic value, along with immune microenvironment correlations, was validated across independent datasets. Furthermore, single-cell transcriptomics was leveraged to investigate cellular heterogeneity, metabolic activities related to TRS, and intercellular communication. Among the TRS genes, *KIF2C* was identified as a tumor-promoting factor. Functional experiments confirmed that *KIF2C* knockdown suppressed LUAD cell growth and invasiveness, underscoring its potential as a target for therapeutic development.

In conclusion, due to its complex and heterogeneous nature, LUAD demands improved stratification frameworks to guide patient-specific treatment planning. By focusing on the underexplored metabolic axis of taurine, our study established a clinically applicable TRS model and identified *KIF2C* as a core oncogenic driver. These results shed light on the regulatory mechanisms underlying LUAD metabolism and immune interactions, supporting future efforts to design therapeutic strategies centered on metabolic pathways.

## 2. Method

### 2.1. Public Dataset Collection

We integrated transcriptomic profiles with clinical characteristics and mutation data of LUAD patients collected from public databases. TCGA RNA-seq data were accessed via the R package TCGAbiolinks, provided in FPKM format, together with clinical annotations and somatic mutation records. The expression matrices were normalized to TPM values for downstream analyses. Six additional LUAD cohorts from GEO were adopted as independent validation datasets. To evaluate predictive capacity under immunotherapy, two immune checkpoint blockade cohorts were included. A detailed summary of all data sources is provided in Supporting Information 1: Table S1. Batch effects across cohorts were adjusted through the sva R package.

To perform single-cell analyses, we adopted two scRNA-seq datasets: GSE171145 from the GEO database and an additional dataset accessed through the CodeOcean platform (https://codeocean.com/capsule/8321305/tree/v1). The GSE171145 dataset includes nine tumor samples from eight LUAD patients, while the CodeOcean dataset comprises 10 normal lung tissues and 10 LUAD tumor samples. All samples were obtained from treatment-naïve individuals and represent primary tumor sites. Detailed clinical and sequencing-related information is available in the original publications and repository annotations. These datasets enabled identification of marker genes specific to individual cell types and characterization of the tumor microenvironment's cellular complexity. A taurine metabolism–associated gene set was compiled according to a previously published study [24], with details provided in Supporting Information 2: Table S2.

To characterize mechanisms of immune evasion, publicly available immune-related datasets were utilized. Immunophenoscore (IPS) values were obtained through The Cancer Immunome Atlas portal, whereas Tumor Immune Dysfunction and Exclusion (TIDE) metrics were accessed from the TIDE platform. All data originated from open-access sources, with prior ethical approval reported in the respective original studies.

### 2.2. Detection of Taurine-Related Gene Coexpression Modules Using WGCNA

Single-sample GSEA (ssGSEA) [25, 26] was performed on TPM-normalized TCGA LUAD RNA-seq profiles to assess the coexpression features of taurine metabolism–related genes. The resulting enrichment scores reflected taurine-associated pathway activity. The resulting enrichment scores for taurine-related pathways were used as trait variables in subsequent WGCNA [27]. The expression matrix was log2-transformed and averaged across replicates, and the 6600 genes ranked highest by median absolute deviation (MAD) were retained. Outlier samples were excluded using hierarchical clustering based on Euclidean distance. The optimal soft-thresholding parameter was identified using the pickSoftThreshold routine, after which a gene adjacency structure was generated and converted into a TOM for hierarchical clustering. Module detection was subsequently performed with cutreeDynamic, setting the minimum cluster size at 200 genes. Highly correlated modules were subsequently merged according to their eigengenes, with a mergeCutHeight parameter set to 0.25, yielding the resulting module classification. To evaluate associations between modules and taurine activity, Pearson correlation coefficients were computed along with corresponding *p*-values between module eigengenes and taurine ssGSEA scores. A heatmap was generated to depict module–trait correlations.

### 2.3. Construction of the TRS

To develop a taurine metabolism-associated prognostic model, three gene sets were integrated: taurine-related genes, coexpression module genes derived from WGCNA, and DEGs obtained by contrasting TCGA LUAD tumors against corresponding normal lung tissues from GTEx. The intersecting genes were analyzed via univariate Cox regression to to evaluate correlations with patient survival.

Subsequently, a multi-algorithmic framework involving 10 distinct models was employed to construct the TRS, including stepwise Cox regression, Lasso, Ridge, CoxBoost, RSF, GBM, elastic net, SuperPC, plsRcox, and survival-SVM. A total of 101 algorithmic configurations underwent repeated tenfold cross-validation. Predictive utility was quantified by the concordance index (C-index) and the TRS version displaying the best average score was adopted as the ultimate model. While informed by previous multi-algorithmic frameworks, our strategy emphasized rigorous internal validation to ensure model stability and predictive strength.

For the final TRS model, CoxBoost and SuperPC algorithms were used in combination. In the CoxBoost model, the penalty parameter was optimized with optimCoxBoostPenalty() (start.penalty = 500) to select penalty values, while the boosting step number (stepno) was tuned through tenfold cross-validation (cv.CoxBoost()), capped at 500 iterations. For SuperPC modeling, superpc.train() was applied with default parameters (e.g., s0.perc = 0.5), and threshold selection relied on the maximum C-index from superpc.cv() across 20 candidate cutoffs using tenfold cross-validation. All computations were performed in R v4.2.1 with CoxBoost (v1.4) and superpc (v1.1).

### 2.4. Evaluation of Risk Stratification and Immunological Features Associated With TRS

After establishing the TRS, the TCGA cohort was partitioned into distinct risk categories, applying the median score as the dividing criterion. An extensive set of evaluations was undertaken to explore how these risk categories relate to patient prognosis, covering [1]: individual risk score distribution [2]; survival outcomes [3]; a heatmap representation of model gene expression. The gene expression matrix underwent *Z*-score normalization and was visualized through hierarchical clustering. To explore the immune landscape differences between the TRS-defined subgroups, the expression of canonical immune checkpoint markers was examined across groups.

In addition, TIDE metrics were accessed via the TIDE platform to assess immune evasion potential, while values obtained from TCIA were used to infer potential responses to immune checkpoint inhibitors. The TRS predictive performance regarding immunotherapy response was validated with two external cohorts: GSE78220, involving melanoma patients undergoing anti–PD-1 therapy [28] and IMvigor210, consisting of patients with metastatic urothelial carcinoma treated with anti–PD-L1 therapy [29]. Patients in each dataset were stratified based on TRS levels and differences in therapeutic responses were analyzed to evaluate the association between TRS and immunotherapy benefit.

### 2.5. Assessment of TRS-Associated Immune Landscape in LUAD

To explore the association between TRS and the structural, as well as functional characteristics of the tumor immune contexture, a comprehensive immunogenomic analysis was carried out. Immune cell infiltration levels in TCGA–LUAD samples were estimated using seven deconvolution tools—TIMER, CIBERSORT, quanTIseq, xCell, EPIC, MCP-counter, and TIDE—via the TIMER2.0 framework [30]. These infiltration patterns were then compared between high-TRS and low-TRS subgroups.

Moreover, stromal and immune indices were derived through the ESTIMATE algorithm to evaluate nonmalignant cell components [31]. TIDE-derived scores were further employed to assess T cell dysfunction and immune exclusion across TRS strata. Finally, IPS data from TCIA were utilized to assess the potential responsiveness of distinct TRS-defined subgroups to immune checkpoint blockade therapy.

### 2.6. Associations Between TRS and Clinical Characteristics With Nomogram Development

To explore the clinical associations of the TRS score, we examined its distribution across demographic and pathological features—including age, sex, and tumor stage—within multiple LUAD patient cohorts. The independent prognostic significance of TRS in the TCGA–LUAD cohort was assessed using univariate and multivariate Cox regression analyses alongside conventional clinical variables. Effect estimates were reported as hazard ratios (HRs) and 95% confidence intervals (CIs). A prognostic nomogram was then developed by integrating TRS with other independent risk indicators to predict survival probabilities at 1-, 3-, and 5-year intervals. To evaluate model performancet, calibration plots were generated to compare predicted outcomes against observed survival, thereby validating the model's accuracy and clinical applicability. In addition, the relationship between TRS and tumor stage was examined by analyzing patient distribution across TRS-defined subgroups, providing preliminary insights into its potential value in clinical risk stratification.

### 2.7. Immune Landscape Profiling via ssGSEA and GSVA

To explore pathway activity variations across different risk groups, multiple enrichment analysis strategies were employed. GSVA was conducted with the GSVA package (v1.46.0) [32], and Hallmark gene collections were obtained from MSigDB via the msigdbr package [33]. TPM-normalized transcriptomic data were preprocessed by averaging replicate genes and excluding genes with mean expression ≤0.5. Pathway-level enrichment scores were derived with the gsva() function and pathway activity differences between high- and low-TRS groups were assessed with the limma package, resulting in a ranked list based on moderated *t*-statistics. To assess the immune landscape, ssGSEA was performed using curated immune-related gene sets (immune.gmt) encompassing immune cells, pathways, and functions. The ssGSEA scores were generated through the ssGSEAParam() and gsva() functions, followed by normalization to a 0–1 range. This allowed the generation of immune infiltration matrices across patients. Subsequently, functional immune signatures (including APC costimulation and Type I/II interferon pathways), along with major immune cell subsets were summarized within TRS categories. Radar plots were constructed using the ggradar package to visualize functional and cellular immune differences between groups. Parameters such as grid scaling, label offset, and color palettes were optimized for clarity. Finally, predefined immunotherapy-related pathways and cancer immune cycle gene sets were analyzed using ssGSEA to evaluate each patient's predicted response to immunotherapy. All enrichment scores were integrated with risk group annotations for comparative analyses.

### 2.8. Single-Cell Atlas Generation and Activity Profiling of Taurine Signatures

All scRNA-seq datasets were analyzed and integrated using the Seurat package (v4.4.0) [34]. Data normalization and identification of highly variable genes were carried out through the NormalizeData and FindVariableFeatures functions, respectively. Mitochondrial and hemoglobin transcript levels were quantified through the PercentageFeatureSet function, and stringent quality control (QC) was implemented based on gene count, UMI abundance, and mitochondrial transcript proportion. Specifically, were retained when meeting the QC thresholds: 500–10,000 detected genes (nFeature_RNA), 1000–100,000 UMI counts (nCount_RNA), mitochondrial transcript fraction (percent.mt) <40%, and hemoglobin gene proportion (percent.HB) <5%. These cutoffs followed overall data distribution patterns and established literature standards to exclude low-quality or multiplet-contaminated cells. Following QC, cell cycle phase inference (CellCycleScoring) and principal component analysis (RunPCA) were conducted. Batch-related variations were addressed using the Harmony algorithm. Cellular clusters were delineated through Louvain-based clustering (FindClusters) and visualized using UMAP (RunUMAP). Cell-type annotation relied on canonical gene markers and prior studies, covering a range of immune and stromal compartments such as dendritic cells and epithelial populations. To evaluate the functional activity of these subsets, taurine-related gene signatures were analyzed using five scoring frameworks: AUCell [35], ssGSEA, AddModuleScore, irGSEA [36], and singscore.

### 2.9. Evaluation of Heterogeneous Communication Patterns Within TME

To examine intercellular communication disparities between high- and low-TRS subgroups, this study performed a systematic analysis of ligand–receptor interaction networks using CellChat (v1.6.1), based on the previously constructed single-cell transcriptomic Seurat object [37]. Cell-type labels and expression profiles derived from the Seurat object were imported into CellChat to generate CellChat objects. Communication probabilities among cell groups were inferred using ligand–receptor interactions curated in the CellChatDB.human reference database. After normalizing the overall interaction strength, significant communication signals were identified through the identifyOverExpressedGenes and computeCommunProb functions, while key signaling pathways and their directional flow were extracted using computeCommunProbPathway and aggregateNet. CellChat objects were independently established for high- and low-TRS subgroups, enabling comparative analysis of intercellular communication networks. The compareInteractions and rankNet functions were used to detect significantly modified pathways and interacting cell subsets. Visualizations such as bubble plots (netVisual_bubble), circle plots (netVisual_circle), and heatmaps were employed to illustrate the intensity and directionality of cell–cell communication.

### 2.10. qRT-PCR for Gene Expression Validation

Total RNA was extracted from A549 and H1299 cells using standard protocols. Reverse transcription was performed to generate cDNA, which was subsequently analyzed by quantitative real-time PCR with SYBR Green chemistry. GAPDH was selected as the internal control, and relative expression levels were determined using the 2^−*ΔΔ*Ct^ method. All experiments were repeated in triplicate to ensure reliability. Primer sequences are provided in Supporting Information 3: Table S3.

### 2.11. Cell Growth Conditions and Transfection Procedures

The human LUAD cell lines A549 (RRID:CVCL_0023) and H1299 (RRID:CVCL_0060) were purchased from the Cell Bank of the Chinese Academy of Sciences (Shanghai, China). A549 cells, derived from a male patient with LUAD, display alveolar epithelial-like characteristics, whereas H1299 cells originate from a lymph node metastasis of a male patient with NSCLC and lack p53 expression. Cells were cultured in RPMI-1640 medium supplemented with 10% fetal bovine serum at 37°C in a humidified incubator containing 5% CO_2_.

For transfection, standard lipid-based reagents were used following established protocols. After transfection, cells were maintained under normal culture conditions for 24–48 h before being harvested for subsequent analyses.

### 2.12. CCK-8 Assay

Cell proliferation capacity was evaluated using a colorimetric assay based on metabolic activity. In brief, A549 and H1299 cells were plated into 96-well culture dishes, and cell viability was monitored at successive time points by adding a substrate solution that produces a measurable color change. The optical density was determined with a microplate reader to reflect relative cell numbers. All measurements were performed in triplicate to ensure consistency.

### 2.13. Transwell Invasion Assay

The invasive potential of LUAD cells was assessed using a chamber-based migration assay with an extracellular matrix coating. Transfected A549 and H1299 cells were suspended in serum-free medium and seeded into the upper compartment, while medium containing serum was placed in the lower compartment to act as a chemoattractant. Following incubation, cells that failed to penetrate the membrane were removed, and those that migrated through were fixed, stained, and enumerated microscopically in several randomly chosen fields. All assays were independently repeated three times to ensure robustness of the results.

### 2.14. Colony Formation Assay

Clonogenic capacity was assessed through a plate-based assay. A small number of A549 and H1299 cells were dispersed into culture wells and allowed to grow under routine conditions until visible colonies had developed. At the end of the incubation period, the colonies were fixed, stained to enhance visualization, and subsequently enumerated under a microscope. Colonies were considered valid only when they exceeded a defined cell number threshold. Each experiment was carried out in three independent replicates to ensure reliability.

### 2.15. Data Processing and Statistical Testing

R software (version 4.2.1) served as the platform for all statistical evaluations. For continuous variables, comparisons between groups were conducted with either Student's *t*-test or the Wilcoxon rank-sum test, depending on data distribution. Categorical variables were analyzed using the chi-square test or Fisher's exact test, as appropriate. Survival curves were estimated with the Kaplan–Meier method, and differences between groups were evaluated by the log-rank test. Univariate and multivariate Cox regression models were applied to identify independent prognostic factors, with results expressed as HRs and 95% CIs. Correlation analyses were carried out using Pearson or Spearman methods according to data normality.

## 3. Results

### 3.1. Taurine Score-Associated Coexpression Modules Highlight Prognostic Markers in LUAD

**Figure 1:**
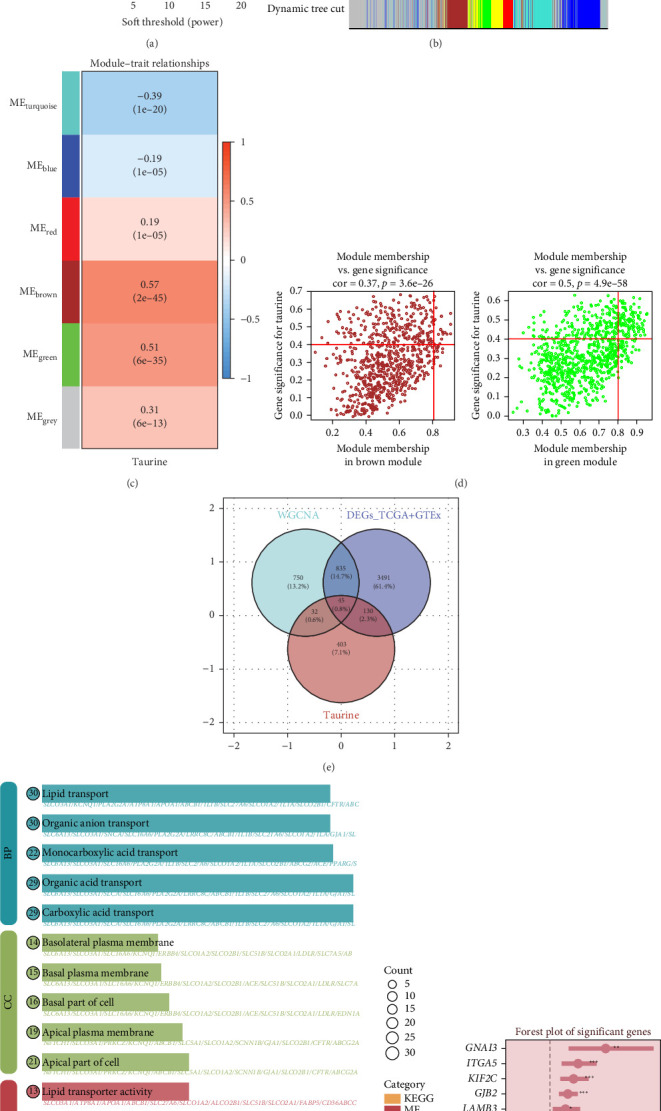
Workflow for WGCNA–based module detection and key gene filtering. (a) An appropriate soft-thresholding value was determined according to scale-free topology criteria (top) and mean connectivity (bottom) across a range of powers. (b) Genes were hierarchically clustered into modules, with distinct colors indicating different co-expression clusters. (c) Module–trait relationships were visualized via a correlation heatmap, illustrating the correlations of module eigengenes with taurine-related scores and corresponding *p*-values. (d) Association between gene-level metrics and module-level significance was assessed by plotting module membership (MM) against gene significance (GS) for representative modules (brown and green). (e) Overlapping genes were identified across key WGCNA modules, DEGs, and taurine metabolism-related gene sets using a Venn diagram. (f) Functional characterization of these intersected genes. (g) Prognostic relevance was evaluated using univariate Cox regression.

To identify pivotal genes associated with TRSs, we applied the WGCNA algorithm to transcriptomic data from TCGA–LUAD. Based on the scale-free topology criterion, a soft-thresholding power of 9 was selected to ensure reliable network construction (Figure 1a,b). This analysis generated multiple gene modules, among which the brown and green clusters exhibited strong correlations with the taurine score and were, therefore, prioritized for downstream exploration (Figure 1c,d). Within these modules, correlations between module eigengenes and gene significance were examined, demonstrating that the clustered genes closely reflected taurine-related transcriptional features. Because of the limited number of normal lung samples in TCGA, RNA-seq data from GTEx were incorporated to strengthen the differential expression comparison. Genes showing pronounced differences between tumor and normal tissues were intersected with the taurine-associated WGCNA modules, yielding 45 candidate genes (Figure 1e). Subsequently, these candidates were annotated by GO and KEGG enrichment analyses, revealing functional involvement in pathways linked to cell adhesion, metabolic remodeling, and immune regulation (Figure 1f). Finally, univariate Cox regression was performed to assess the prognostic implications of these genes, and several were identified as being significantly related to overall survival, including both protective and risk-associated factors (Figure 1g).

### 3.2. Establishment of the TRS Model and Robustness Assessment Across Multiple Cohorts

**Figure 2:**
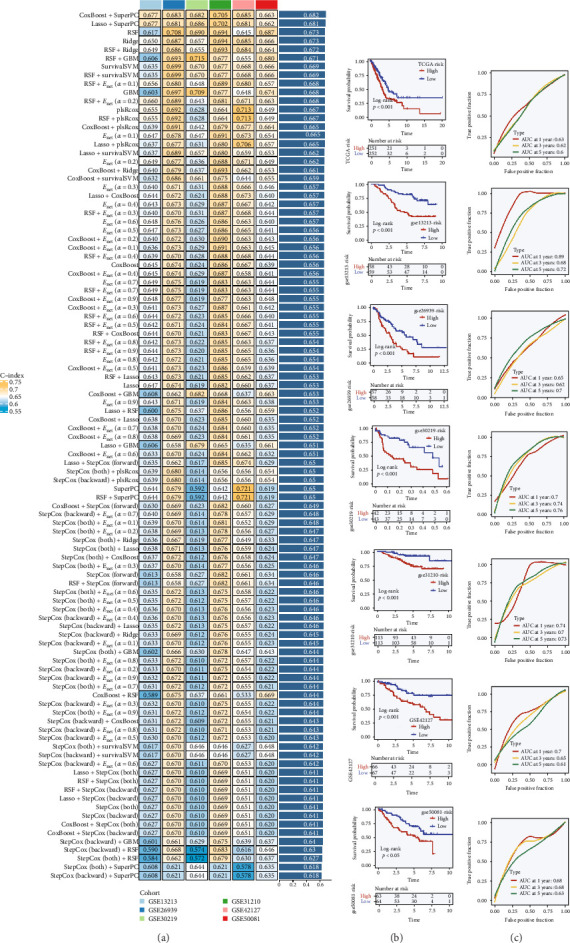
Construction and evaluation of the TRS scoring model. (a) A comprehensive modeling framework was applied by integrating 10 machine learning algorithms in a pairwise fashion, resulting in 101 unique TRS prediction models. Their predictive concordance was quantified using the C-index in the TCGA–LUAD dataset, with performance indicated by a color-coded scale. (b) The optimal model combination (CoxBoost and SuperPC) was selected based on highest concordance and further evaluated via Kaplan–Meier survival curves across the TCGA–LUAD cohort and six external datasets. (c) Time-dependent ROC analysis was conducted to assess the prognostic discrimination of the TRS signature at 1, 3, and 5 years in multiple cohorts, with AUC values annotated for each time point.

To establish a reliable prognostic framework, we systematically combined 10 commonly used machine learning strategies—including CoxBoost, SuperPC, Lasso, RSF, Enet, and Stepwise–Cox—resulting in 101 distinct modeling schemes. Each model was tested within the TCGA–LUAD training cohort and predictive efficiency was assessed using the C-index. The approach yielding the greatest C-index was retained as the final TRS model (Figure 2a). While most methods demonstrated reasonable predictive capacity (C-index > 0.6), the CoxBoost algorithm, particularly when integrated with SuperPC, consistently showed superior performance and was therefore applied in subsequent analyses.

Using the optimized CoxBoost + SuperPC framework, TRS values were computed for every patient, and cases were stratified into high- and low-risk groups on the basis of the median score. Kaplan–Meier analysis in the TCGA cohort indicated that patients with elevated TRS values experienced significantly poorer survival outcomes (*p*  < 0.001). The prognostic separation was further corroborated in six independent GEO cohorts, each confirming stable risk group discrimination (Figure 2b). In addition, time-dependent ROC curves were generated, demonstrating high AUC values at 1-, 3-, and 5-year intervals across both training and validation datasets, highlighting the robustness and broad applicability of the TRS model (Figure 2c).

### 3.3. TRS Score Reflects Immune State and Predicts Response to Immune Checkpoint Therapy

To further clarify the immunological significance of the TRS score, we systematically examined its relationship with TME and potential response to ICIs.

**Figure 3:**
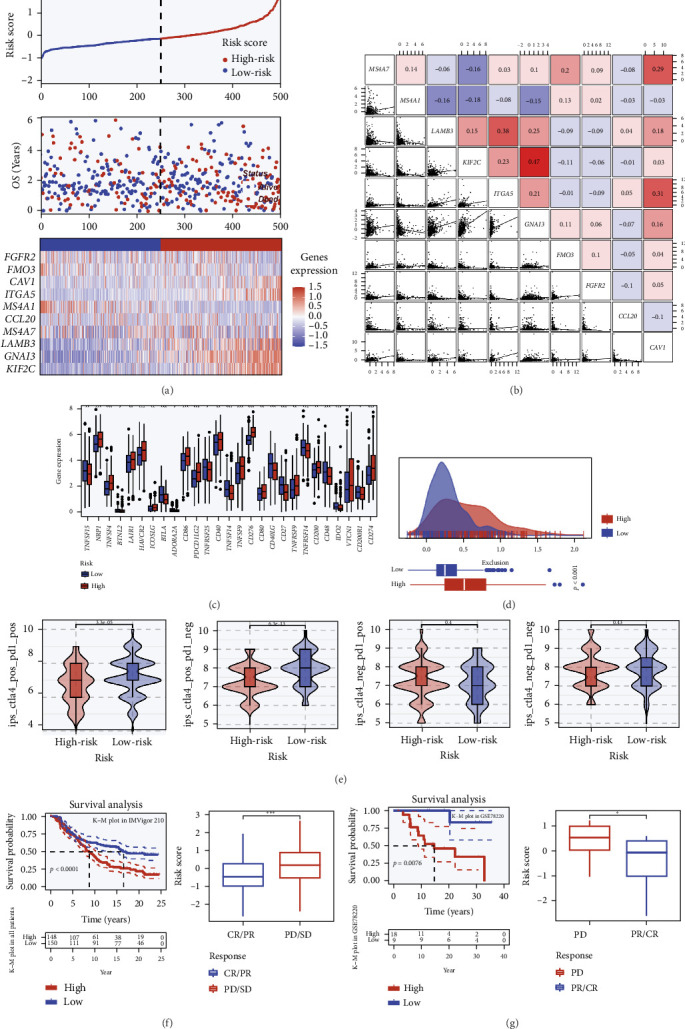
Association between TRS score, immune microenvironment features, and immunotherapy response. (a) Survival risk plot based on the TRS score. (b) Coexpression correlation matrix of TRS model genes in the TCGA–LUAD cohort, with Pearson correlation coefficients indicated. (c) Comparison of immune checkpoint gene expression across high- and low-TRS cohorts. (d) Comparison of the exclusion scores from the TIDE database between TRS-defined groups to evaluate immune evasion potential. (e) Immune-related scores (IPSs) from the TCIA database assessing predicted responsiveness to anti-CTLA4 and anti-PD1 therapies across TRS subgroups. (f) Prognostic value of the TRS score in the IMvigor210 immunotherapy cohort, including Kaplan–Meier survival analysis and comparisons of TRS scores across treatment response groups (CR/PR vs. SD/PD). (g) Validation of the immunotherapy predictive capacity of the TRS score in the GSE78220 cohort, with survival analysis and response-based TRS score comparisons.

As illustrated in Figure 3a, the risk curve and scatter plot indicated that patients with high TRS scores faced a markedly increased mortality risk. Expression heatmaps of representative model genes revealed distinct patterns between high- and low-risk subgroups, suggesting that elevated TRS was associated with an immunosuppressive phenotype (Figure 3b). Correlation analyses of model genes demonstrated strong positive intergene associations, implying coordinated regulation. Evaluation of immune checkpoint molecules (Figure 3c) showed that several inhibitory receptors were upregulated in the high-TRS subgroup, consistent with an immunosuppressive immune profile. The complete list of checkpoint-related genes included in the analysis is provided in Supporting Information 4: Table S4. TIDE analysis (Figure 3d) further revealed that Exclusion scores were significantly higher in the high-TRS group (*p*  < 0.01), reinforcing the link between TRS and an immunosuppressive tumor context. When stratified by TRS level (Figure 3e), patients with higher scores exhibited substantially increased TIDE prediction values, particularly under PD-1/PD-L1- and CTLA4-related ICB models (*p*  < 0.05). These results support the notion that TRS may act as a surrogate marker for immune resistance under specific therapeutic conditions. External validation in two independent immunotherapy cohorts (Figure 3f) confirmed the predictive role of TRS. In the IMvigor210 dataset, patients with low TRS scores experienced significantly improved survival, and TRS was markedly lower in individuals achieving complete or partial responses compared to those with stable or progressive disease. A similar pattern was seen in the GSE78220 cohort (Figure 3g), where TRS stratification clearly separated responders (CR/PR) from nonresponders (SD/PD). Collectively, these findings demonstrate that the TRS score is tightly linked to immune checkpoint activity and serves as an indicator of both immune state and immunotherapy responsiveness.

### 3.4. TRS Score Reflects Distinct Patterns of Immune Infiltration and Regulation

**Figure 4:**
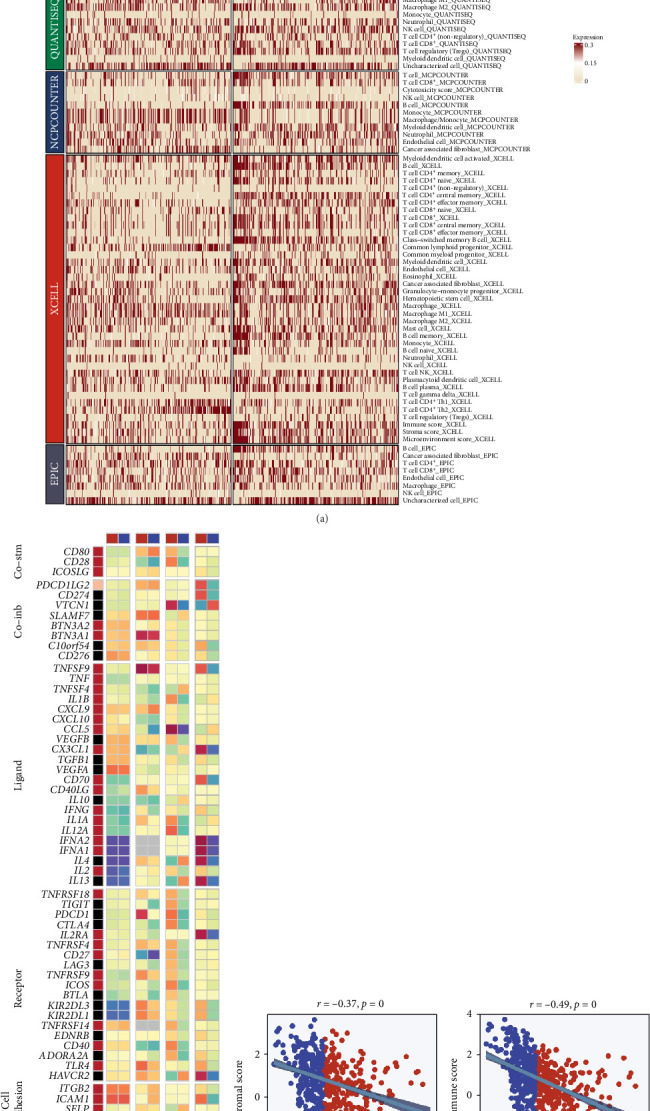
TRS score is closely linked to immune microenvironment features in LUAD. (a) Comparative heatmap illustrating the abundance of immune cell subsets between high- and low-TRS groups. Infiltration scores were derived from seven established immune deconvolution algorithms. Rows correspond to immune cell types, with red indicating enriched and blue indicating reduced infiltration. (b) Differential expression profiles of immune regulatory genes—including costimulatory/inhibitory markers, ligands, receptors, antigen presentation machinery, and adhesion molecules—across TRS subgroups. Expression levels are color-coded, while gray indicates genes with undetectable expression. (c) Correlation analysis between TRS scores and immune microenvironment indices estimated via the ESTIMATE algorithm.

To further clarify how the TRS score is linked with the tumor immune landscape, immune cell infiltration was estimated using diverse computational frameworks (Figure 4a). Patients with elevated TRS values demonstrated pronounced reductions in infiltrating effector cells such as CD8^+^ T cells, NK cells, and dendritic cells, reflecting an immune-suppressed phenotype. Subsequent analyses revealed that immune-regulatory molecules were differentially expressed across TRS-defined groups, with activating and inhibitory receptors being more abundant in the low-TRS subgroup (Figure 4b). Genes associated with antigen processing and chemokine signaling were also enriched, implying enhanced immunological activity in these patients. In addition, the ESTIMATE approach was used to quantify stromal and immune components of the microenvironment (Figure 4c). TRS values showed a significant negative correlation with ImmuneScore, StromalScore, and ESTIMATEScore, while displaying a positive relationship with TumorPurity. Together, these findings indicate that high TRS tumors are characterized by immune evasion, diminished stromal participation, and an overall immunosuppressive milieu. Collectively, the TRS score not only serves as a prognostic marker but also encapsulates critical aspects of tumor–immune interactions, underscoring its relevance for immunotherapy guidance.

### 3.5. Clinical Associations and Prognostic Significance of the TRS Score

**Figure 5:**
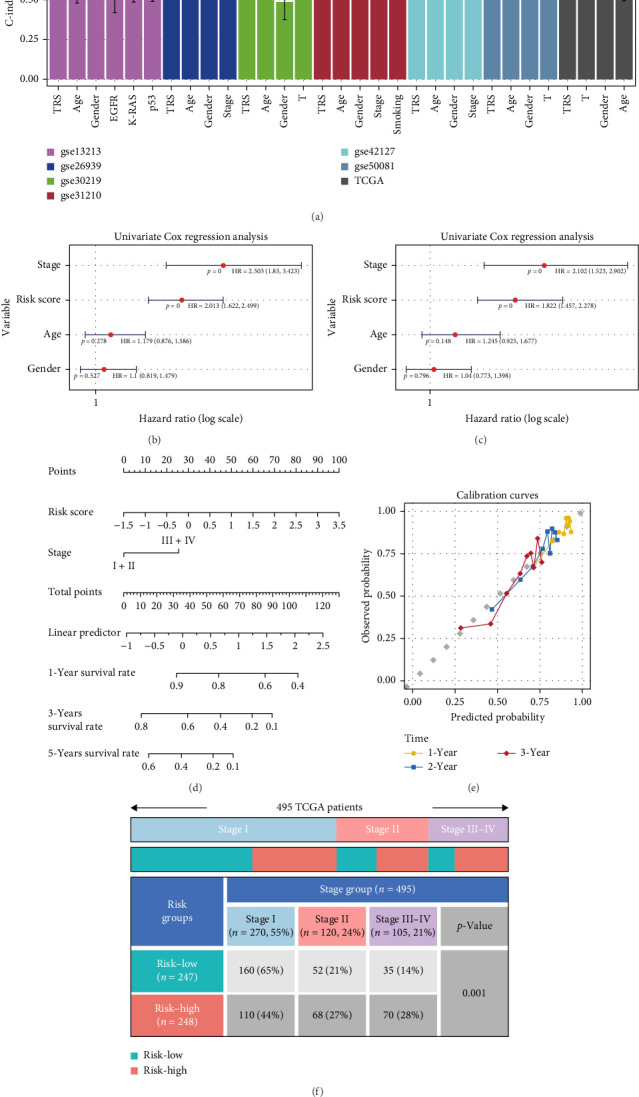
Integration of TRS with clinical variables and prognostic modeling. (a) Comparative evaluation of the concordance index (C-index) for TRS versus conventional clinical factors (age, gender, stage) across the TCGA and six external cohorts. (b) Univariate Cox regression analyses highlighting the prognostic relevance of TRS and clinical variables. (c) Multivariate analysis confirming the TRS score as an independent predictor of overall survival after adjustment for key clinical parameters. (d) Predictive nomogram incorporating TRS alongside significant clinical indicators for estimating 1-, 3-, and 5-year survival probabilities in LUAD patients. (e) Visual inspection of calibration performance showing high concordance between predicted and observed survival outcomes. (f) Risk group stratification by tumor stage, demonstrating a correlation between TRS classification and pathological staging.

To examine the clinical relevance of the TRS score, its distribution across different pathological subgroups was first assessed (Figure 5a). The analysis revealed clear differences in TRS scores among distinct clinical categories, indicating potential value in patient stratification.

Univariate Cox regression in the TCGA–LUAD cohort showed that the TRS score, together with variables such as pathological stage, was significantly associated with overall survival (Figure 5b). Multivariate analysis further demonstrated that both TRS and stage independently retained prognostic significance after adjustment for other clinical factors, underscoring the additional predictive contribution of TRS beyond traditional indicators (Figure 5c).

Based on two independent prognostic indicators—TRS score and pathological stage—a predictive nomogram was developed to estimate survival probability for LUAD patients (Figure 5d). The model assigns weighted points for each factor, and the cumulative score can be translated into the likelihood of survival at 1, 3, and 5 years (Figure 5e). Calibration plots demonstrated close concordance between predicted and actual survival outcomes, supporting the robustness and reliability of this prognostic tool. To further explore its clinical significance, TRS distribution was compared across TNM stages (Figure 5f). Patients with more advanced disease showed significantly elevated TRS values, implying stronger tumor aggressiveness and higher progression risk, thereby underscoring its potential value in clinical stratification.

### 3.6. TRS Score Reflects Functional Stratification of the Immune Microenvironment

**Figure 6:**
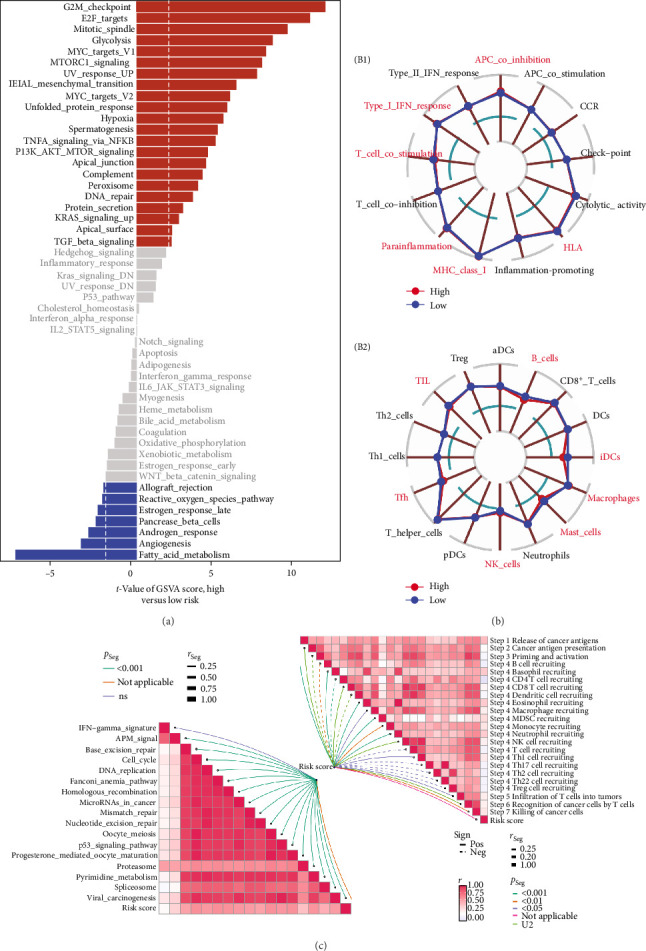
Enrichment analysis reveals immunological features associated with TRS score. (a) Pathway enrichment analysis based on GSVA. The bar plot displays pathways significantly enriched in the high- versus low-TRS groups, ranked by *t*-values. Red bars indicate pathways upregulated in the high-risk group, while blue bars represent those enriched in the low-risk group. (b) Radar plots showing differences between high and low TRS groups in terms of immune cell infiltration (B1) and immune-related functional activities (B2). Each axis represents a specific immune cell type or immune function. (c) Immune cycle activity analysis. The left panel highlights immune processes significantly altered between groups. The right heatmap shows correlations and significance levels between different steps of the cancer-immunity cycle. Color intensity indicates the degree of correlation, and dot size reflects the statistical significance (*p*-value).

GSVA was applied to explore functional differences associated with TRS levels. The analysis revealed that high-TRS patients exhibited elevated activity in hallmark pathways linked to cell cycle progression and cellular stress, including the G2M checkpoint, E2F target activation, spindle assembly, DNA repair mechanisms, mTOR signaling, glycolytic metabolism, and apoptotic processes. Conversely, immune-metabolic programs such as fatty acid oxidation, oxidative phosphorylation, antiviral defense, and interferon signaling were predominantly enriched in the low-TRS subgroup (Figure 6a).

Using the ssGSEA method, immune composition and functional signatures were further delineated. Radar plot analysis showed that patients with low TRS values exhibited markedly greater infiltration of multiple immune subsets, such as T follicular helper cells, cytotoxic CD8^+^ T cells, natural killer cells, and antigen-presenting dendritic cells. This immunological enrichment was accompanied by enhanced cytotoxic gene programs, amplified type II interferon activity, and stronger engagement of anti-inflammatory signaling pathways (Figure 6b).

Analysis within the cancer–immunity cycle framework revealed that tumors classified with low TRS values displayed heightened activity across several phases of immune activation, including antigen processing, T-cell initiation, immune cell trafficking, and cytotoxic responses. These findings indicate that this subgroup exhibits a more vigorous antitumor immune profile (Figure 6c). Overall, TRS appears to act not only as a prognostic indicator but also as a marker reflecting immune activation dynamics within the tumor microenvironment.

### 3.7. Integration of Five Scoring Algorithms Highlights Suppressed Taurine Metabolism in Tumor Tissues

**Figure 7:**
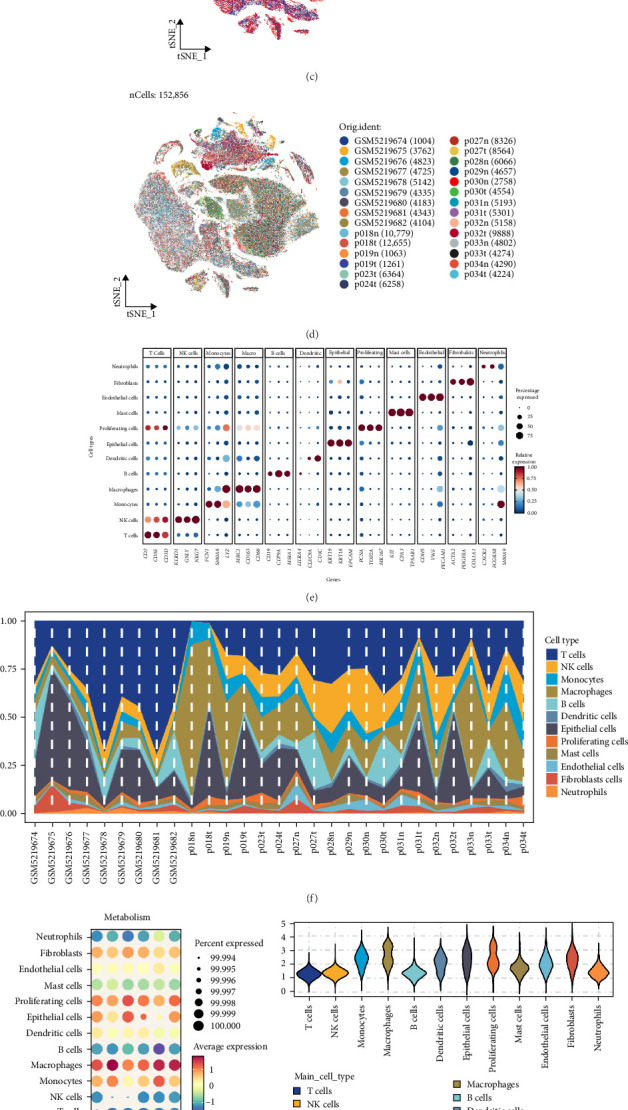
Single-cell analysis reveals cellular composition and taurine metabolism activity characteristics. (a) t-SNE plot displaying the clustering results of 35 distinct cell populations. (b) Cell type annotation based on canonical marker genes, identifying 12 major cell lineages. (c) Spatial distribution of cells from different tissue origins. (d) Distribution of cells from individual samples across the transcriptomic landscape. (e) Dot plot illustrating the expression level and percentage of typical marker genes across different cell types. (f) Proportional abundance of various cell types. (g) Dot plot showing taurine pathway activity scores calculated using five algorithms: AUCell, AddModuleScore, ssGSEA, singscore, and UCell. (h) Violin plot demonstrating the distribution of taurine metabolism scores across cell types. (i) Comparison of taurine metabolism activity scores between tumor and normal tissues.

Following stringent QC (see Supporting Information 5: Figure S1 for QC metrics), a total of 152,856 high-quality single cells were retained for subsequent analysis. A comprehensive LUAD single-cell atlas was generated, comprising 35 distinct cellular clusters (Figure 7a). Using the expression profiles of canonical marker genes, these clusters were categorized into 12 representative immune and stromal lineages. These included lymphoid subsets (T cells, B cells, and NK cells) and myeloid populations (monocytes, macrophages, dendritic cells, neutrophils, and mast cells), as well as nonimmune components such as epithelial cells, proliferating cells, fibroblasts, and endothelial cells (Figure 7b). Comparison of cellular distribution between tumor and adjacent normal tissues revealed distinct compositional differences (Figure 7c), while marked heterogeneity was observed across individual samples (Figure 7d,f).

To further investigate taurine-related metabolic activity across cell populations, five independent computational scoring methods (AUCell, AddModuleScore, ssGSEA, singscore, and UCell) were applied. Results consistently indicated that nonimmune lineages, such as epithelial cells, proliferating cells, and fibroblasts, exhibited higher taurine-associated metabolic activity, whereas immune subsets including T cells, B cells, and NK cells showed lower scores (Figure 7g,h). Moreover, cells derived from tumor samples displayed significantly reduced taurine metabolic activity compared with those from normal counterparts (Figure 7i), suggesting suppression of taurine metabolism within the tumor microenvironment.

### 3.8. Association Between Taurine Metabolism and Cell–Cell Interactions in TME

**Figure 8:**
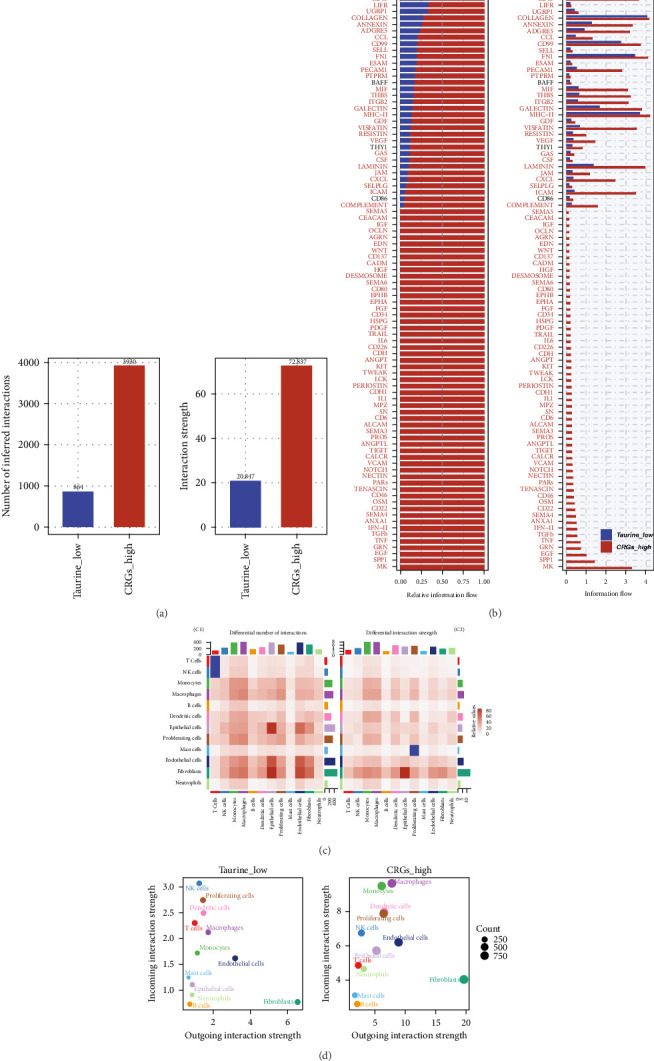
Intergroup variation in signaling networks associated with taurine metabolism status revealed by CellChat. (a) Overview of intercellular communication. (b) Comparison of signaling pathway activity. Subpart B1 illustrates the relative information flow of each signaling pathway, while Subpart B2 shows the total information flow within each group. (c) Heatmaps depicting cell–cell interaction differences. (C1) Heatmap indicates the variation in the number of interactions, and (C2) heatmap shows differences in interaction strength. Red indicates enhanced signaling in the CRGs_high group, while blue indicates stronger communication in the Taurine_low group. (d) Outgoing and incoming signaling strength of each cell type in the two groups. Each dot represents a cell type, and the size of the dot corresponds to the number of cells.

Building on the constructed single-cell atlas, we systematically compared intercellular communication patterns between the high- and low-TRS subgroups (Figure 8a). The analysis revealed that both the overall number and strength of inferred ligand–receptor interactions were significantly elevated in the high-TRS subgroup, suggesting that enhanced taurine metabolic activity may be accompanied by more active cellular cross talk. We next assessed the directional flow of signaling pathways across subgroups. Several key pathways, including MK, SPP1, CD22, and IL1, demonstrated stronger outgoing signaling in the high-TRS group, highlighting their potential regulatory roles under high-metabolism conditions (Figure 8b). Heatmap visualization further illustrated marked differences in intercellular communication among distinct cell types. In particular, interaction frequency and intensity between immune-related cells (e.g., endothelial cells and fibroblasts) and epithelial cells were notably increased in the high-TRS group, implying enhanced microenvironmental modulation (Figure 8c). Finally, we calculated the incoming and outgoing interaction strengths for each cell type. Proliferating cells, macrophages, and fibroblasts in the high-TRS subgroup displayed markedly higher levels of signaling activity within the communication network (Figure 8d). Collectively, these findings indicate that tumors with elevated TRS values harbor more complex and dynamic intercellular communication landscapes, potentially driving immune regulation and tumor progression.

### 3.9. Exploring Personalized Therapeutic Strategies Guided by TRS Stratification

Drug response profiling was performed between TRS-defined risk groups using the oncoPredict algorithm. As illustrated in Supporting Information 6: Figure S2A, the low-TRS subgroup exhibited reduced predicted sensitivity (higher IC50 estimates) to several standard chemotherapeutic agents, such as Paclitaxel, 5-Fluorouracil, Topotecan, Erlotinib, and Dasatinib. In contrast, patients with high TRS scores showed increased resistance signatures toward multiple targeted therapies, including Sorafenib, Rapamycin, Temsirolimus, Lapatinib, and Axitinib (Supporting Information 6: Figure S2B). These differential drug response patterns may offer valuable insights for tailoring individualized treatment approaches in LUAD.

### 3.10. *KIF2C* Is Overexpressed in LUAD With Experimental Evidence Supporting Its Oncogenic Role

**Figure 9:**
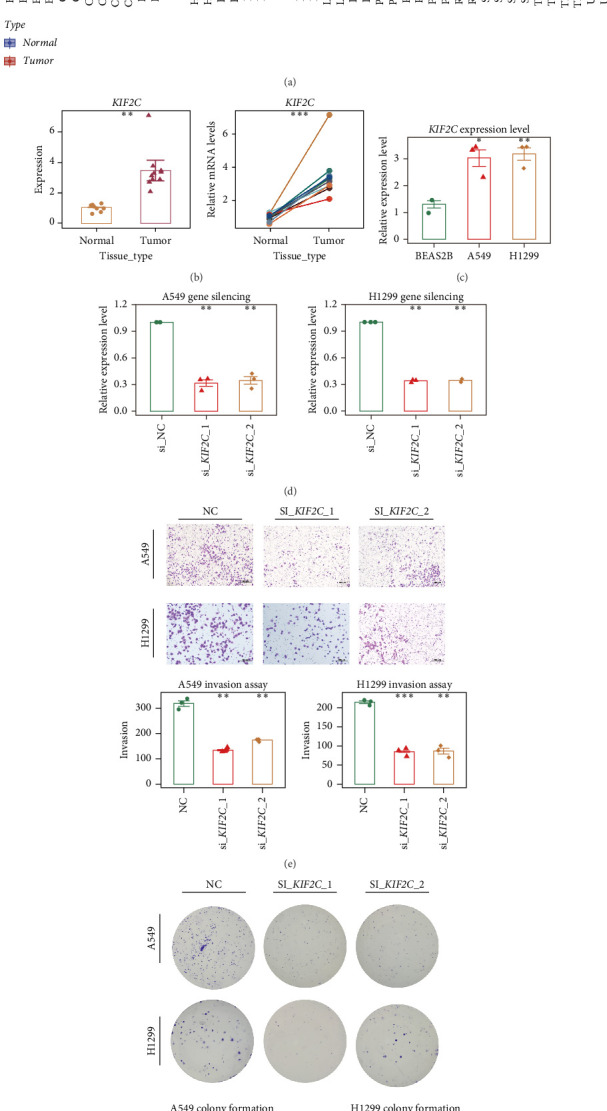
Expression analysis and functional assays of *KIF2C*. (a) Expression distribution of *KIF2C* across different tissue types in the TCGA pan-cancer dataset. (b) qRT-PCR validation of *KIF2C* expression in clinical LUAD samples from Tianjin Chest Hospital, including overall comparison and matched pairs. (c) mRNA expression levels of *KIF2C* in BEAS-2B, A549, and H1299 lung cell lines. (d) Efficiency of *KIF2C* knockdown in A549 and H1299 cells after transfection with si_*KIF2C*_1 and si_*KIF2C*_2. (e) Transwell assays showing migration patterns of A549 and H1299 cells under different conditions, along with quantitative analysis. (f) Colony formation assays illustrating colony growth in A549 and H1299 cells across groups, with corresponding quantification.

As an initial step, TCGA pan-cancer transcriptomic data were mined to characterize the expression landscape of *KIF2C* and its potential implications in LUAD. The analysis demonstrated that *KIF2C* was markedly upregulated in multiple cancer types, with particularly strong elevation observed in LUAD (Figure 9a). To further assess its prognostic relevance in a broader cancer context, pan-cancer survival analyses were performed, revealing that *KIF2C* expression was significantly correlated with overall survival and progression-related outcomes, including OS, DFS, DSS, and PFS (Supporting Information 7: Figure S3). Validation using clinical samples from Tianjin Chest Hospital confirmed that *KIF2C* expression levels were significantly higher in tumor tissues compared with matched adjacent non-cancerous tissues, as determined by quantitative PCR (Figure 9b). At the cellular level, LUAD lines A549 and H1299 exhibited elevated *KIF2C* mRNA expression compared to the normal bronchial epithelial line BEAS-2B (Figure 9c). For functional interrogation, siRNAs targeting *KIF2C* were transfected into A549 and H1299 cells, with efficient knockdown verified by qRT-PCR (Figure 9d). Silencing of *KIF2C* resulted in a pronounced reduction of invasive ability, as assessed by transwell assays (Figure 9e), and a marked decrease in colony formation capacity (Figure 9f). Collectively, these findings support a tumor-promoting role of *KIF2C* in LUAD, likely through enhancing proliferative and invasive properties of cancer cells.

## 4. Discussion

LUAD, the most common subtype of NSCLC, represents a major global health concern because of its high frequency and associated mortality [3, 4]. Despite advances in molecular classification, the introduction of targeted agents and immune-based treatments that have prolonged patient survival in certain subpopulations, substantial challenges remain, such as delayed detection, pronounced intratumoral heterogeneity, and multifaceted resistance mechanisms [38]. In this context, accurate patient stratification, prognostic evaluation, and prediction of treatment response have become central issues in LUAD research.

Reprogramming of cellular metabolism is widely acknowledged as a defining feature of cancer. Taurine, a sulfur-containing amino acid broadly distributed in human tissues, plays essential roles in osmoregulation, antioxidant defense, inflammatory response, and immune modulation [17, 21, 23]. Emerging studies have implicated taurine metabolism in tumor progression across multiple malignancies, potentially influencing energy remodeling, apoptosis resistance, and immune microenvironment reshaping [39]. Therefore, systematic investigation of taurine-related metabolic genes in LUAD holds promise for elucidating novel metabolic features, identifying prognostic biomarkers, and guiding therapeutic innovation.

In this study, we integrated transcriptomic data from TCGA and GEO cohorts, aiming to build a taurine-related risk score (TRS) using cross-cohort data. Using multiple machine learning algorithms, we developed a prognostic model that stratified LUAD patients into discrete risk groups. The TRS–based classifier showed consistent predictive performance across validation datasets. In addition, we further examined links between TRS and clinical/molecular characteristics, including immune infiltration, genomic instability, and predicted response to checkpoint therapy. High TRS aligned with an immune-cold microenvironment, increased TMB, and reduced benefit from immunotherapy, supporting TRS as a biomarker for individualized management.

Among the genes comprising the TRS signature, we centered on *KIF2C*, also known as MCAK, a kinesin-13 microtubule depolymerase essential for spindle integrity and chromosome segregation [40, 41]. *KIF2C* is frequently overexpressed across solid tumors and drives unchecked cell-cycle progression, genomic instability, and activation of PI3K/AKT and Wnt/β-catenin signaling [42, 43]. In our study, we identified *KIF2C* as markedly upregulated in LUAD, positively correlated with TRS, and indicative of worse prognosis. Functional assays revealed *KIF2C* knockdown curtailed proliferation, invasion, and clonogenic growth, suggesting its tumor-promoting role downstream of taurine metabolism. As a key gene within the TRS framework, *KIF2C* represents a promising therapeutic target for metabolic intervention in LUAD.

In conclusion, a prognostic index associated with taurine metabolism was developed and systematically tested in LUAD patients by leveraging transcriptomic data from multiple independent cohorts and employing machine learning algorithms. This study underscores the importance of metabolic remodeling centered on taurine in shaping LUAD progression and immune dynamics. Additionally, *KIF2C* was pinpointed as a potential mediator linking metabolic alterations to tumor aggressiveness, with implications for future therapeutic intervention. Collectively, these results offer an alternative framework for risk classification and provide new avenues for personalized treatment strategies in LUAD.

Nevertheless, our study has several limitations. Although cross-validation was conducted across several external cohorts, validation in larger clinical datasets is required to establish the robustness and generalizability of the TRS model. In addition, the predictive association between TRS scores and immunotherapy response requires further validation in real-world immunotherapy-treated cohorts. Mechanistically, the role of *KIF2C* remains incompletely understood and warrants deeper investigation through in vivo studies and clinical specimen analysis.

Future studies should explore the stratification potential of TRS in combination therapy settings and investigate the interplay between taurine metabolism and other metabolic pathways. These insights provide a foundation for designing metabolism-oriented therapies and individualized management strategies in LUAD.

## Data Availability

All data used in this study are retrieved from publicly available sources. The specific repositories are listed in the manuscript. Further details can be obtained by contacting the corresponding author.

## Supporting Information

Supporting Information 1 Table S1. Datasets analyzed in this research.

Supporting Information 2 Table S2. Gene set associated with taurine metabolism.

Supporting Information 3 Table S3. siRNA sequences and primers used for KIF2C.

Supporting Information 4 Table S4: Checkpoint-related gene.

Supporting Information 5 Figure S1. Quality control and batch effect assessment of scRNA-seq data. Violin plots of gene number, UMI count, and pMT before (A) and after (B) quality control across all samples. PCA plots illustrating sample distribution and batch effect before (C) and after (D) quality filtering. PCA projections of cell cycle phases. (E) Before cell cycle regression and (F) after cell cycle effect was regressed out.

Supporting Information 6 Figure S2. Drug response prediction according to TRS classification. (A) Example drugs with lower IC50 estimates in the low-risk TRS subgroup, implying enhanced sensitivity. (B) Example drugs with higher IC50 estimates in the high-risk TRS subgroup.

Supporting Information 7 Figure S3. Prognostic significance of KIF2C across multiple cancer types. (A–D) Pan-cancer survival analyses of KIF2C based on TCGA cohorts, showing its associations with OS, DFS, DSS, and PFS.
