# Supplementary figures and images for: A Machine Learning-Derived Taurine Metabolism Signature Predicts Prognosis and Immune Landscape in Lung Adenocarcinoma via Integrative Single-Cell Analysis
Source: Mediators Inflamm. 2025 Nov 25;2025:6610564. doi: 10.1155/mi/6610564 (PMC12672081; doi:10.1155/mi/6610564)

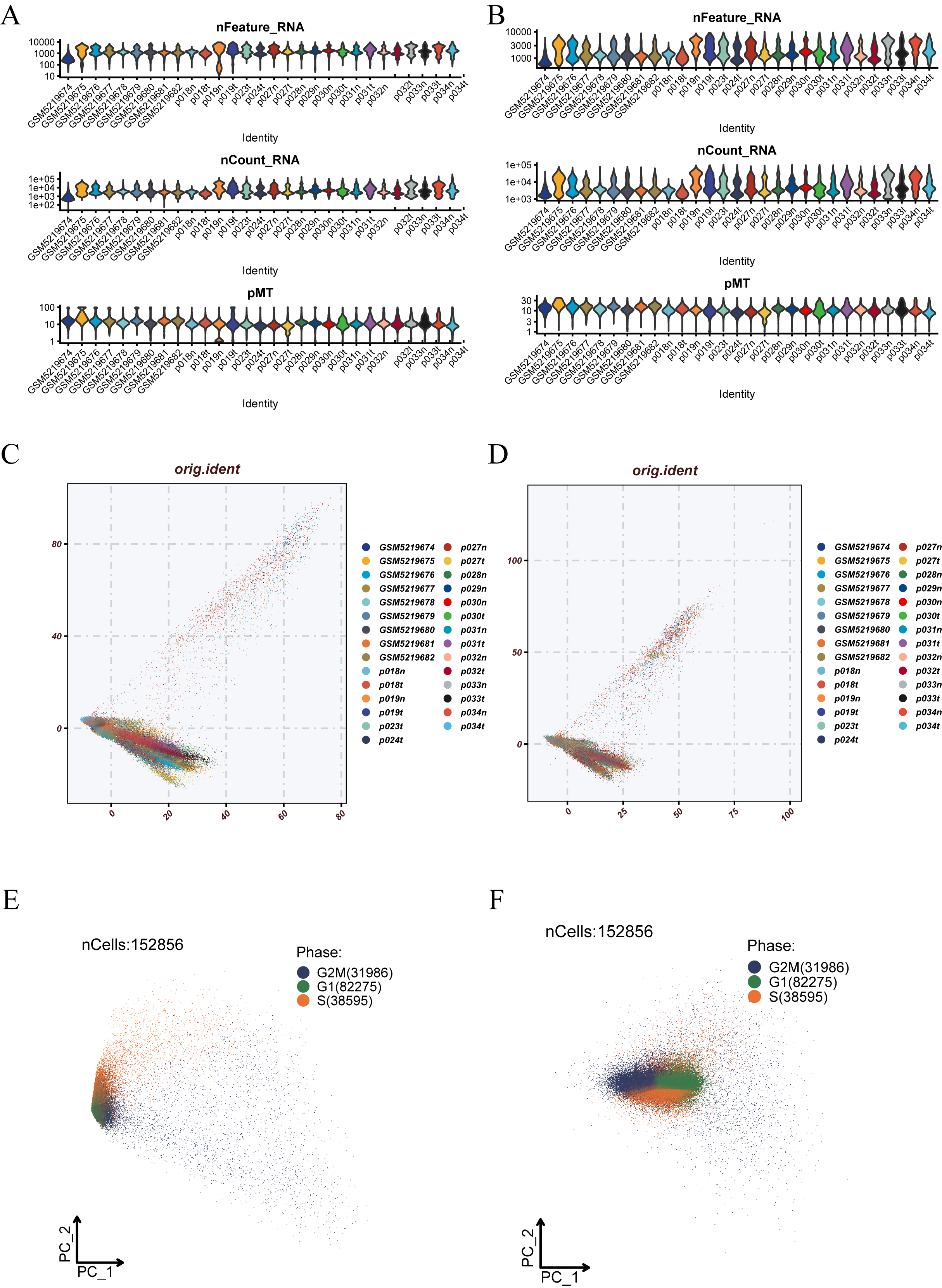

Supplement: Supporting Information 5 — Figure S1. Quality control and batch effect assessment of scRNA-seq data. Violin plots of gene number, UMI count, and pMT before (A) and after (B) quality control across all samples. PCA plots illustrating sample distribution and batch effect before (C) and after (D) quality filtering. PCA projections of cell cycle phases. (E) Before cell cycle regression and (F) after cell cycle effect was regressed out. [file 6610564.f5.tif]

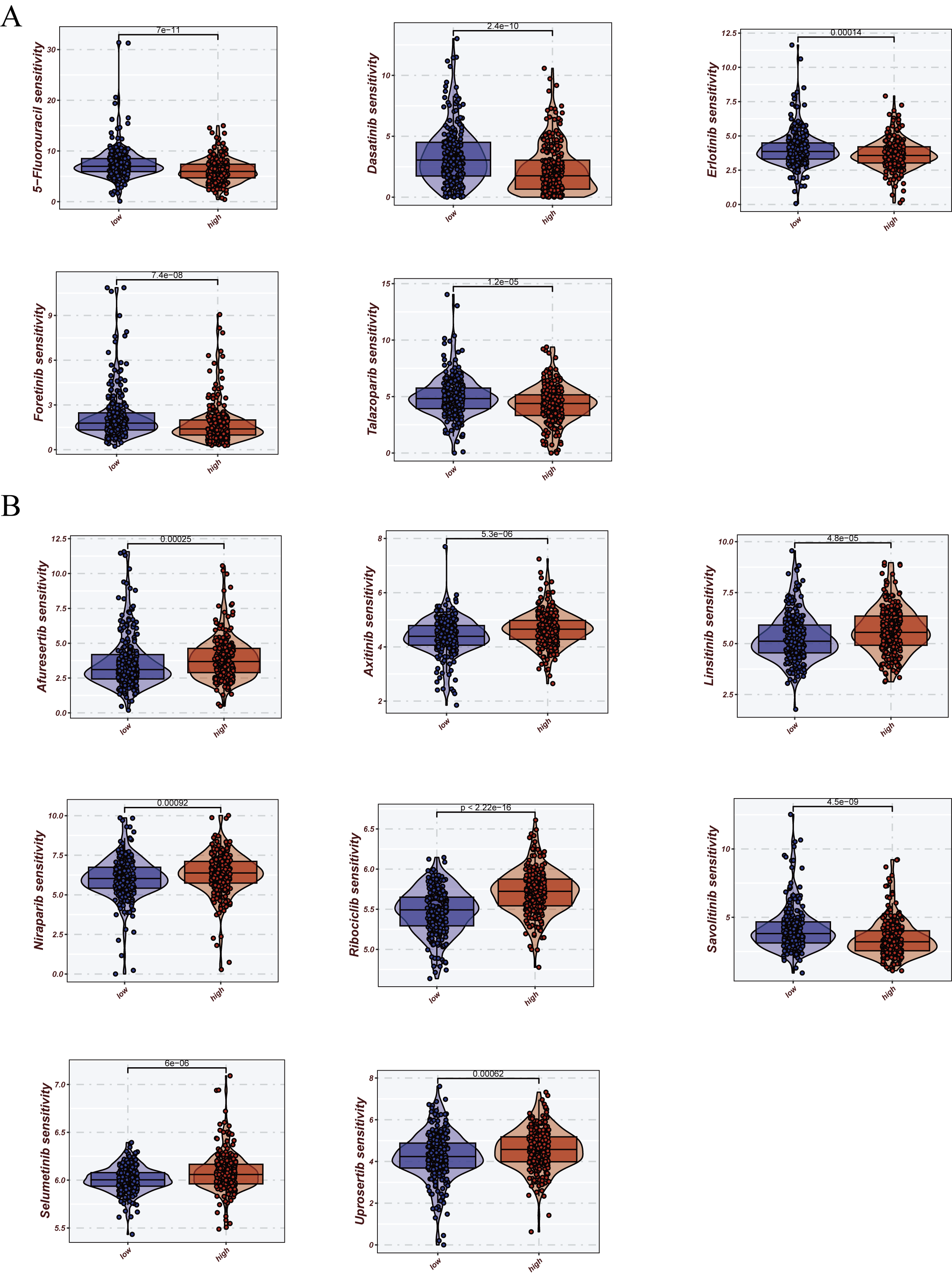

Supplement: Supporting Information 6 — Figure S2. Drug response prediction according to TRS classification. (A) Example drugs with lower IC50 estimates in the low-risk TRS subgroup, implying enhanced sensitivity. (B) Example drugs with higher IC50 estimates in the high-risk TRS subgroup. [file 6610564.f6.tif]

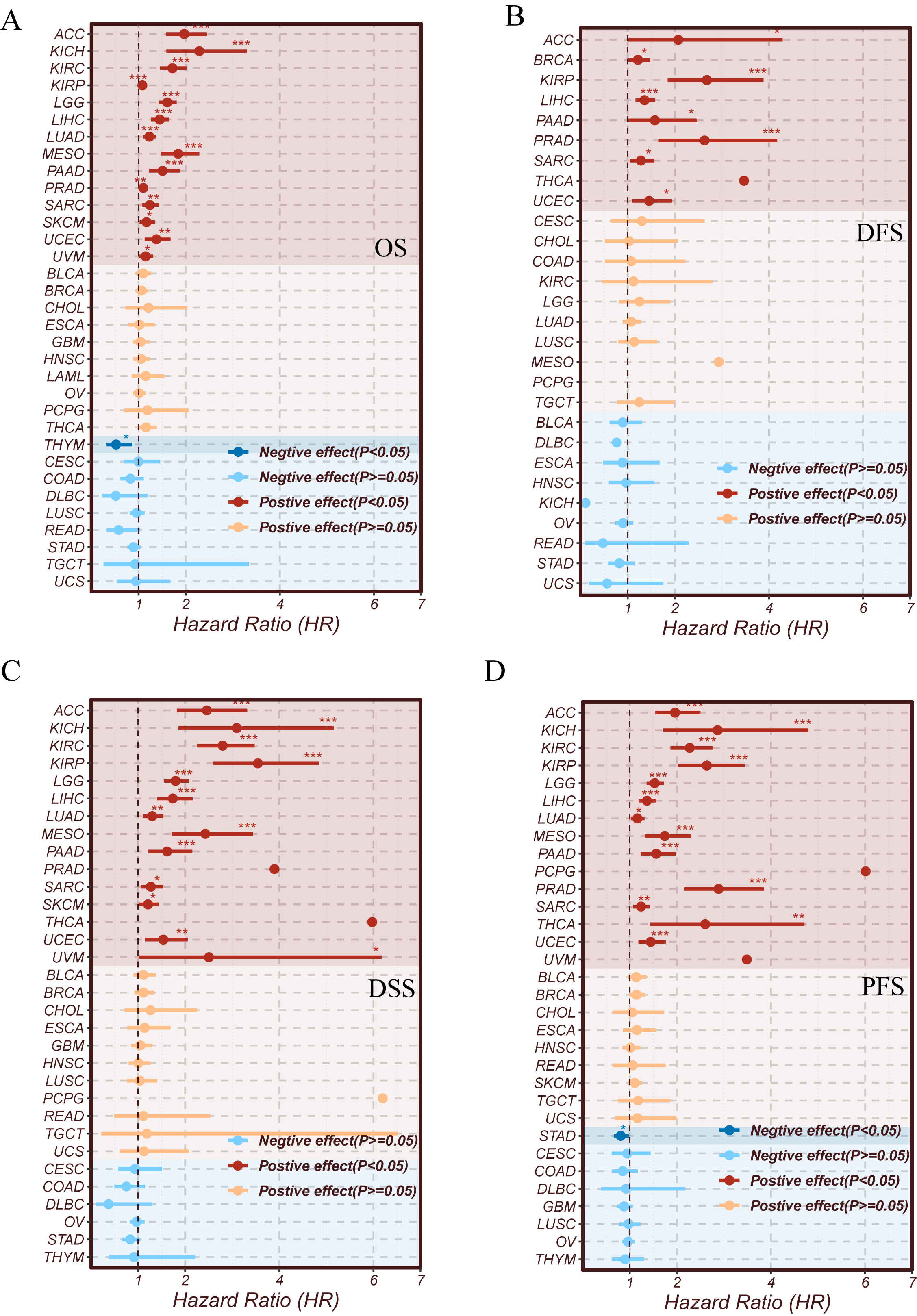

Supplement: Supporting Information 7 — Figure S3. Prognostic significance of KIF2C across multiple cancer types. (A–D) Pan-cancer survival analyses of KIF2C based on TCGA cohorts, showing its associations with OS, DFS, DSS, and PFS. [file 6610564.f7.tif]
